# Supplementary material for: Genomic and Biochemical Characterization of Bifidobacterium pseudocatenulatum JCLA3 Isolated from Human Intestine
Source: Microorganisms. 2022 Oct 22;10(11):2100. doi: 10.3390/microorganisms10112100 (PMC9695335; doi:10.3390/microorganisms10112100)
Supplement: Supplementary file 1 [file microorganisms-10-02100-s001.zip › microorganisms-1958389-supplementary.pdf]

Supplementary Materials:

Table S1: *Bifidobacterium pseudocatenulatum* encoded enzymes.

Class	Subclasses	EC	Number of enzymes found
**Transferases**	Transfer one carbon group	2.1	26
	transfer aldehyde or ketonic groups	2.2	5
	acyltransferases	2.3	21
	glycosyltransferases	2.4	22
	transferring alkyl or aryl groups other than methyl groups	2.5	14
	transferring nitrogenous groups	2.6	10
	transferring phosphorus-containing groups	2.7	82
	transferring sulfur-containing groups	2.8	4
**Hydrolases**	acting on ester bonds	3.1	44
	Glycosylases	3.2	46
	Peptidases	3.4	32
	acting on carbon-nitrogen bonds, other than peptide bonds	3.5	23
	acting on acid anhydrides	3.6	32
	acting on halide bonds	EC 3.8.1.2	1
**Oxidoreductases**	acting on the CH-OH group of donors	1.1	29
	aldehyde or oxo group of donors	1.2	5
	acting on the CH-CH group of donors	1.3	6
	acting on the CH-NH_2 group of donors	1.4	**3**
	acting on the CH-NH group of donors	1.5	**5**
	acting on NADH or NADPH	1.6	**3**
	acting on a sulfur group of donors	1.8	**3**
	acting on diphenols and related substances as donors	1.10	**1**
	acting on a peroxide as acceptor	1.11	**2**
	acting on CH or CH_2 groups	1.17	**5**
	acting on iron-sulfur proteins as donors	1.18	**1**
	acting on phosphorus or arsenic in donors	1.20	**2**
	other oxidoreductases	1.97	2
**Ligases**	forming carbon-oxygen bonds	6.1	20
	forming carbon-sulfur bonds	6.2	6
	forming carbon-nitrogen bonds	6.3	35
	forming carbon-carbon bonds	6.4	2
	forming phosphoric-ester bonds	6.5	1
**Lyases**	carbon-carbon lyases	4.1	13
	carbon-oxygen lyases	4.2	19
	carbon-nitrogen lyases	4.3	9

	carbon-sulfur lyases	4.4	4
	phosphorus-oxygen lyases	4.6	2
**Isomerases**	Racemases and epimerases	5.1	9
	cis-trans-Isomerases	5.2	1
	intramolecular oxidoreductases	5.3	8
	intramolecular transferases	5.4	13
	intramolecular lyases	5.5	1
	isomerases altering macromolecular conformation	5.6	6
**Translocases**	Catalyzing the translocation of hydrons	7.1	2
	catalyzing the translocation of inorganic cations	7.2	3
	Catalyzing the translocation of inorganic anions and it chelates	7.3	1
	catalyzing the translocation of amino acids and peptides	7.4	7
	translocation of carbohydrates and their derivatives	7.5	2
	translocation of other compounds	7.6	3

Table S2: *Bifidobacterium pseudocatenulatum´s* genes associated with carbohydrate metabolism, their respective enzymes, and the possible pathway in which they play a role according to the KEGG database.

B. pseudocatenulatum **JCLA3 locus_tag**	**Gene_Bif_KEGG**	**Protein name**	**Pathway**
L0J99_00020	BBPC_1812	alpha-galactosidase	Galactose metabolism Glycerolipid metabolism Sphingolipid metabolism
L0J99_00060	BBPC_1818	glycosyl hydrolase	Starch and sucrose metabolism Cyanoamino acid metabolism
L0J99_00240	BBPC_0011	L-lactate dehydrogenase	Glycolysis / Gluconeogenesis Pyruvate metabolism Propanoate metabolism Cysteine and methionine metabolism Exosome
L0J99_00255	BBPC_0015	phosphoenolpyruvate carboxylase	Pyruvate metabolism Methane metabolism
L0J99_00285	BBPC_0021	glycogen phosphorylase	Starch and sucrose metabolism

L0J99_00450	BBPC_0057	fructose-bisphosphate aldolase	Glycolysis / Gluconeogenesis Pentose phosphate pathway Fructose and mannose metabolism Methane metabolism
L0J99_00480	BBPC_0063	sucrose phosphorylase	Starch and sucrose metabolism
L0J99_01450	BBPC_0270	glycosyl hydrolase	Starch and sucrose metabolism
L0J99_01750	BBPC_0326	4-alpha-glucanotransferase	Starch and sucrose metabolism
L0J99_01765	BBPC_0329	glycosyl hydrolase	Starch and sucrose metabolism
L0J99_03885	BBPC_0749	glycosyl hydrolase	Starch and sucrose metabolism
L0J99_04125	BBPC_0806	Pullulanase	Starch and sucrose metabolism
L0J99_08150	BBPC_1479	glycoside hydrolase	Starch and sucrose metabolism
L0J99_08195	BBPC_1479	glycoside hydrolase	Starch and sucrose metabolism
L0J99_09820	BBPC_1721	putative pullulanase	Starch and sucrose metabolism
L0J99_09845	BBPC_1726	4-alpha-glucanotransferase	Starch and sucrose metabolism
L0J99_00490	BBPC_0065	glycosyl hydrolase	Galactose metabolism Starch and sucrose metabolism
L0J99_00880	BBPC_0155	2-isopropylmalate synthase	Pyruvate metabolism Valine, leucine, and isoleucine biosynthesis
L0J99_00935	BBPC_0166	myo-inositol-1-phosphate synthase	Inositol phosphate metabolism
L0J99_01065	BBPC_0188	isopropylmalate isomerase large subunit	C5-Branched dibasic acid metabolism Valine, leucine, and isoleucine biosynthesis
L0J99_01070	BBPC_0189	isopropylmalate isomerase small subunit	C5-Branched dibasic acid metabolism Valine, leucine, and isoleucine biosynthesis
L0J99_01130	BBPC_0207	6-phosphogluconate dehydrogenase-like protein	Pentose phosphate pathway Glutathione metabolism
L0J99_01140	BBPC_0209	carbohydrate kinase	Pentose phosphate pathway

L0J99_01225	BBPC_0226	acetolactate synthase small subunit	Butanoate metabolism C5-Branched dibasic acid metabolism<br>Valine, leucine, and isoleucine biosynthesis<br>Pantothenate and CoA biosynthesis
L0J99_01230	BBPC_0227	acetolactate synthase large subunit	Butanoate metabolism C5-Branched dibasic acid metabolism<br>Valine, leucine, and isoleucine biosynthesis<br>Pantothenate and CoA biosynthesis
L0J99_01315	BBPC_0244	glucose-6-phosphate isomerase	Glycolysis / Gluconeogenesis Pentose phosphate pathway Starch and sucrose metabolism<br>Amino sugar and nucleotide sugar metabolism
L0J99_01430	BBPC_0266	propionyl-CoA carboxylase alpha subunit	Pyruvate metabolism Glyoxylate and dicarboxylate metabolism<br>Propanoate metabolism Fatty acid biosynthesis Valine, leucine, and isoleucine degradation
L0J99_01435	BBPC_0267	propionyl-CoA carboxylase beta subunit	Glyoxylate and dicarboxylate metabolism<br>Propanoate metabolism Valine, leucine, and isoleucine degradation
L0J99_01770	BBPC_0330	Lyase	Pyruvate metabolism
L0J99_01785	BBPC_0333	dehydrogenase	Pentose and glucuronate interconversions
L0J99_01795	BBPC_0335	aldehyde-alcohol dehydrogenase	Glycolysis / Gluconeogenesis Pyruvate metabolism Butanoate metabolism Fatty acid degradation Tyrosine metabolism Chloroalkane and chloroalkene degradation Naphthalene degradation
L0J99_02000	BBPC_0376	ribose-5-phosphate isomerase	Pentose phosphate pathway

L0J99_02020	BBPC_0380	phosphoglucomutase	Glycolysis / Gluconeogenesis Pentose phosphate pathway Galactose metabolism Starch and sucrose metabolism Amino sugar and nucleotide sugar metabolism Purine metabolism
L0J99_02025	BBPC_0381	PTS system glucose-specific IIABC components	Phosphotransferase system (PTS) Transporters
L0J99_02120	BBPC_0405	Phosphoglyceromutase	Glycolysis / Gluconeogenesis Methane metabolism Glycine, serine, and threonine metabolism Membrane trafficking
L0J99_02275	BBPC_0429	putative oxidoreductase	Glyoxylate and dicarboxylate metabolism Propanoate metabolism
L0J99_02410	BBPC_0457	xylulose kinase	Pentose and glucuronate interconversions
L0J99_02525	BBPC_0457	xylulose kinase	Pentose and glucuronate interconversions
L0J99_02410	BBPC_0475	xylulose kinase	Pentose and glucuronate interconversions
L0J99_02525	BBPC_0475	xylulose kinase	Pentose and glucuronate interconversions
L0J99_02450	BBPC_0466	xylose isomerase	Pentose and glucuronate interconversions Fructose and mannose metabolism
L0J99_01695	BBPC_0315	beta-1	Amino sugar and nucleotide sugar metabolism
L0J99_02380	BBPC_0315	beta-1	Amino sugar and nucleotide sugar metabolism
L0J99_02480	BBPC_0315	beta-1	Amino sugar and nucleotide sugar metabolism
L0J99_02455	BBPC_0467	glycosyl hydrolase	Amino sugar and nucleotide sugar metabolism
L0J99_01695	BBPC_0472	beta-1	Amino sugar and nucleotide sugar metabolism
L0J99_02380	BBPC_0472	beta-1	Amino sugar and nucleotide sugar metabolism
L0J99_02480	BBPC_0472	beta-1	Amino sugar and nucleotide sugar metabolism
L0J99_09635	BBPC_1686	N-acetylglucosamine-6-phosphate deacetylase	Amino sugar and nucleotide sugar metabolism

L0J99_09640	BBPC_1687	glucosamine-6-phosphate deaminase	Amino sugar and nucleotide sugar metabolism
L0J99_02630	BBPC_0495	citrate synthase	Citrate cycle (TCA cycle) Glyoxylate and dicarboxylate metabolism
L0J99_02695	BBPC_0509	D-fructose-6-phosphate amido transferase	Amino sugar and nucleotide sugar metabolism Alanine, aspartate, and glutamate metabolism Peptidases and inhibitors
L0J99_02710	BBPC_0515	beta-galactosidase	Galactose metabolism
L0J99_02970	BBPC_0568	alcohol dehydrogenase	Glycolysis / Gluconeogenesis Pyruvate metabolism Fatty acid degradation Tyrosine metabolism Chloroalkane and chloroalkene degradation Naphthalene degradation
L0J99_03140	BBPC_0601	putative inositol monophosphatase	Inositol phosphate metabolism
L0J99_03440	BBPC_0660	succinate dehydrogenase iron-sulfur subunit	Citrate cycle (TCA cycle) Butanoate metabolism Oxidative phosphorylation
L0J99_03445	BBPC_0661	succinate dehydrogenase flavoprotein subunit	Citrate cycle (TCA cycle) Butanoate metabolism Oxidative phosphorylation
L0J99_03675	BBPC_0707	6-phosphogluconate dehydrogenase	Pentose phosphate pathway Glutathione metabolism
L0J99_03695	BBPC_0710	glucose-6-phosphate 1-dehydrogenase	Pentose phosphate pathway Glutathione metabolism
L0J99_03705	BBPC_0712	6-phosphogluconolactonase	Pentose phosphate pathway
L0J99_03745	BBPC_0720	putative B-exosaminidase	Amino sugar and nucleotide sugar metabolism beta-Lactam resistance
L0J99_03770	BBPC_0725	Enolase	Glycolysis / Gluconeogenesis Methane metabolism RNA degradation Messenger RNA biogenesis
L0J99_03940	BBPC_0760	pyruvate kinase	Glycolysis / Gluconeogenesis Pyruvate metabolism Membrane trafficking

L0J99_03950	BBPC_0762	3-hydroxyacyl-CoA dehydrogenase	Butanoate metabolism Phenylalanine metabolism Benzoate degradation
L0J99_03955	BBPC_0763	conserved hypothetical protein	Glycolysis / Gluconeogenesis
L0J99_04015	BBPC_0784	xylulose-5-phosphate/fructose-6-phosphate phosphoketolase	Pentose phosphate pathway
L0J99_04020	BBPC_0785	phosphate acetyltransferase	Pyruvate metabolism Propanoate metabolism Methane metabolism Taurine and hypotaurine metabolism
L0J99_04025	BBPC_0786	acetate kinase	Pyruvate metabolism Propanoate metabolism Methane metabolism Taurine and hypotaurine metabolism
L0J99_04170	BBPC_0815	glucose-1-phosphate adenylyltransferase	Starch and sucrose metabolism<br>Amino sugar and nucleotide sugar metabolism
L0J99_04220	BBPC_0824	NAD(P) transhydrogenase alpha-1 subunit	Nicotinate and nicotinamide metabolism
L0J99_04225	BBPC_0825	NAD(P) transhydrogenase alpha-2 subunit	Nicotinate and nicotinamide metabolism
L0J99_04230	BBPC_0826	NAD(P) transhydrogenase beta subunit	Nicotinate and nicotinamide metabolism
L0J99_04355	BBPC_0852	glutamate synthase beta subunit	Nitrogen metabolism Alanine, aspartate, and glutamate metabolism
L0J99_04365	BBPC_0854	putative glycosyltransferase	Starch and sucrose metabolism<br>Glycosyltransferases
L0J99_04520	BBPC_0884	isocitrate dehydrogenase	Citrate cycle (TCA cycle) Glutathione metabolism
L0J99_04585	BBPC_0897	putative phosphoglycerate mutase	Glycolysis / Gluconeogenesis Methane metabolism Glycine, serine, and threonine metabolism<br>Membrane trafficking

L0J99_05025	BBPC_0936	triosephosphate isomerase	Glycolysis / Gluconeogenesis Fructose and mannose metabolism<br>Inositol phosphate metabolism
L0J99_05065	BBPC_0944	transaldolase	Pentose phosphate pathway
L0J99_05070	BBPC_0945	transketolase	Pentose phosphate pathway
L0J99_05230	BBPC_0961	UTP-glucose-1-phosphate uridylyltransferase	Pentose and glucuronate interconversions<br>Galactose metabolism Starch and sucrose metabolism<br>Amino sugar and nucleotide sugar metabolism
L0J99_05275	BBPC_0968	succinyl-CoA synthase alpha subunit	Citrate cycle (TCA cycle) Propanoate metabolism C5-Branched dibasic acid metabolism
L0J99_05355	BBPC_0984	ribulose-phosphate 3-epimerase	Pentose phosphate pathway Pentose and glucuronate interconversions
L0J99_05705	BBPC_1057	dihydrolipoamide dehydrogenase	Glycolysis / Gluconeogenesis<br>Citrate cycle (TCA cycle)<br>Pyruvate metabolism<br>Glyoxylate and dicarboxylate metabolism<br>Propanoate metabolism<br>Glycine, serine, and threonine metabolism<br>Valine, leucine, and isoleucine degradation<br>Lysine degradation<br>Tryptophan metabolism
L0J99_05770	BBPC_1070	putative glucokinase	Glycolysis / Gluconeogenesis Amino sugar and nucleotide sugar metabolism
L0J99_05970	BBPC_1111	formate acetyltransferase	Pyruvate metabolism Propanoate metabolism Butanoate metabolism
L0J99_07615	BBPC_1372	putative beta-glucosidase	Starch and sucrose metabolism<br>Cyanoamino acid metabolism
L0J99_07635	BBPC_1376	putative beta-glucosidase	Starch and sucrose metabolism<br>Cyanoamino acid metabolism

L0J99_07625	BBPC_1374	glycerate kinase	Glyoxylate and dicarboxylate metabolism<br>Glycerolipid metabolism<br>Glycine, serine, and threonine metabolism
L0J99_08650	BBPC_1580	ATP synthase beta subunit	Oxidative phosphorylation<br>Photosynthesis proteins
L0J99_08660	BBPC_1582	ATP synthase alpha subunit	Oxidative phosphorylation<br>Photosynthesis proteins
L0J99_08750	BBPC_1592	putative amylase	Starch and sucrose metabolism
L0J99_08755	BBPC_1593	inorganic pyrophosphatase	Oxidative phosphorylation
L0J99_09250	BBPC_1743	beta-galactosidase	Galactose metabolism<br>Sphingolipid metabolism<br>Other glycan degradation
L0J99_09385	BBPC_1743	beta-galactosidase	Galactose metabolism<br>Sphingolipid metabolism<br>Other glycan degradation
L0J99_09685	BBPC_1743	beta-galactosidase	Galactose metabolism<br>Sphingolipid metabolism<br>Other glycan degradation
L0J99_09350	BBPC_1761	glycosyl hydrolase	Starch and sucrose metabolism<br>Cyanoamino acid metabolism
L0J99_09250	BBPC_1768	beta-galactosidase	Galactose metabolism<br>Sphingolipid metabolism<br>Other glycan degradation
L0J99_09385	BBPC_1768	beta-galactosidase	Galactose metabolism<br>Sphingolipid metabolism<br>Other glycan degradation
L0J99_09685	BBPC_1768	beta-galactosidase	Galactose metabolism<br>Sphingolipid metabolism<br>Other glycan degradation
L0J99_09475	BBPC_1786	putative thioredoxin reductase	Seleno compound metabolism
L0J99_09555	BBPC_1670	alpha-galactosidase	Galactose metabolism<br>Glycerolipid metabolism<br>Sphingolipid metabolism
L0J99_09650	BBPC_1689	glucokinase	Glycolysis / Gluconeogenesis<br>Galactose metabolism<br>Starch and sucrose metabolism<br>Amino sugar and nucleotide sugar metabolism

L0J99_09250	BBPC_1695	beta-galactosidase	Galactose metabolism Sphingolipid metabolism Other glycan degradation
L0J99_09385	BBPC_1695	beta-galactosidase	Galactose metabolism Sphingolipid metabolism Other glycan degradation
L0J99_09685	BBPC_1695	beta-galactosidase	Galactose metabolism Sphingolipid metabolism Other glycan degradation
L0J99_09460	BBPC_1782	oligo-1	Galactose metabolism Starch and sucrose metabolism
L0J99_09945	BBPC_1782	oligo-1	Galactose metabolism Starch and sucrose metabolism
L0J99_09840	BBPC_1725	glycosyl hydrolase	Galactose metabolism Starch and sucrose metabolism
L0J99_09880	BBPC_1736	putative alpha-1	Galactose metabolism Starch and sucrose metabolism
